# Qualitative methods in the development of a parent survey of children’s oral health status

**DOI:** 10.1186/s41687-018-0033-x

**Published:** 2018-03-01

**Authors:** Carl A. Maida, Marvin Marcus, Ron D. Hays, Ian D. Coulter, Francisco Ramos-Gomez, Steve Y. Lee, Patricia S. McClory, Laura V. Van, Yan Wang, Jie Shen, Bryant Lau, Vladimir W. Spolsky, James J. Crall, Honghu Liu

**Affiliations:** 1 School of Dentistry, Division of Public Health & Community Dentistry and Division of Oral Biology & Medicine, University of California, Los Angeles, Box 951668, 10833 Le Conte Avenue, Los Angeles, CA 90095-1668 USA; 2 School of Dentistry, Division of Public Health & Community Dentistry, University of California, Los Angeles, Los Angeles, CA 90095 USA; 3 Department of Medicine, Division of General Internal Medicine and Health Services Research, University of California, Los Angeles, Los Angeles, USA; 4 Department of Health Policy and Management, Fielding School of Public Health, Los Angeles, CA 90095 USA; 5 RAND Corporation, Santa Monica, CA 90401 USA; 6 School of Dentistry, Division of Growth & Development, Section of Pediatric Dentistry, University of California, Los Angeles, Los Angeles, CA 90095 USA; 7 School of Dentistry, Division of Constitutive & Regenerative Sciences, Section of Restorative Dentistry, University of California, Los Angeles, Los Angeles, CA 90095 USA; 8 School of Dentistry, University of California, Los Angeles, Los Angeles, CA 90095 USA; 9 Department of Biostatistics, Fielding School of Public Health, Los Angeles, CA 90095 USA; 10 College of Letters and Science, University of California, Los Angeles, Los Angeles, CA 90095 USA; 11 Department of Medicine, Division of General Internal Medicine and Health Services Research, Los Angeles, CA 90095 USA

**Keywords:** Child and adolescent oral health, Outcomes, Psychosocial aspects of oral health, Qualitative research

## Abstract

**Background:**

Parents’ perceptions of their 8–17-year-old children’s oral health status were assessed using a sample from diverse dental clinics in Greater Los Angeles County to identify constructs for a survey instrument.

**Methods:**

Focus groups with 29 parents or guardians were conducted to identify themes that informed development of survey items. The draft items were administered to a different group of 32 parents or guardians in cognitive interviews, and revised for subsequent field-testing.

**Results:**

Thematic and narrative analyses were performed after the focus groups and key lay-oriented dimensions were uncovered, notably the relationship between oral health, systemic health and the life course. In the cognitive interviews, parents entered multiple responses to questions related to the look of their child’s teeth, and their overall perception of tooth color. Parents also assessed their child’s fear or discomfort with the dental experience, and other social and psychological concerns related to oral health status. The temporal dimensions of certain items were specified; for example, oral pain and mood items were revised to include duration of the symptom or mood state. As parents tended to confuse oral health maintenance and prevention, these two related concepts were separated into two items. Based on the qualitative work, we revised items in preparation for a field test.

**Conclusions:**

As a PRO measurement study, qualitative research informed a field test survey to assess factors associated with oral health status and the individual’s perceptions and subjective views of these constructs for eventual item development for epidemiological and clinical use.

## Background

This study builds upon two previous efforts: the development of the Children’s Oral Health Status Index (COHSI) [1], a clinically oriented outcome measure, and the Patient-Reported Outcome Measurement Information System (PROMIS®) project [2]. The COHSI is a measure derived from paired preferences of an expert panel of dentists. PROMIS® was initiated by the National Institute of Health in 2004 to develop state-of-the-science self-reported health measures, such as: pain, fatigue, physical functioning emotional distress, and social role participation. While PROMIS® item banks have been developed for child and adolescent health they do not target child and adolescent oral health [3]*.* This study uses the methods employed by PROMIS® to develop oral health patient-reported outcome (PRO) items. The intent of our oral health study is to relate both the child’s and the parent’s reports about the child’s oral health with clinical assessments of oral health status by dental examiners. In the study’s initial phases, qualitative approaches from PROMIS® were used to develop PRO items that address clinical oral health status, a clinical outcome, rather than relying solely on perceptions of oral health status or oral health-related quality of life (Oral HRQol). Qualitative research in our study provides the bridge between lay perceptions and the clinician. In this paper, we report on qualitative research centered on understanding parents’ perceptions of oral health with the objective of developing survey items that can estimate clinical measures of oral health status.

Assessing child and adolescent oral health outcomes is complex because of changes associated with emerging-developmental skills and functions [4–6]. Middle childhood (ages 8–12 years) is a life stage characterized by learning cognitive skills, gaining competence in interpersonal and social relationships, and acquiring habits of mind essential for more focused learning and work tasks. It is also a time when children, while still influenced by their family, experience a strong peer orientation and spend considerable energy cultivating and maintaining friendships. Hence, the main developmental task of middle childhood is one of the integration of inner life within a complex social world in order to build a foundation to meet upcoming adolescent challenges. Adolescents, by contrast, are faced with two main developmental tasks: to integrate and adapt to the physiological changes within themselves and to prepare themselves for the tangible adult tasks ahead of them. Our study population for this paper, children and adolescents, ages 8–17, also experiences transitional and permanent dentition, and relatively high rates of dental and occlusal problems. Understanding parent perceptions of oral health status for older children and youth is especially important as their influence on oral hygiene, as with other aspects of their children’s development, begins to diminish as the child becomes more enmeshed into the social world beyond the family.

Existing measures to assess children’s perceptions include the Child Oral Health Impact Profile [7] and Child Oral Health-related Quality of Life [8] measures. Self-reports by children are the primary method of oral health status and oral HRQoL assessment, supplemented by parent or guardian proxy report [9, 10]. However, beyond these self-reports by children and their parents, a number of issues remain to be addressed in PRO assessment, including evaluation of the conceptual frameworks guiding oral health assessment and the psychometric properties of the most widely used measures [11]*.*

A systematic review indicated that parents’ reports of their children’s health provide substantial unique information beyond their children’s self reports [12]. Another study comparing single-item parental ratings of children’s oral health with clinical indicators [13] found significant associations of parent ratings of their child’s oral health with children’s self-reports and clinical indicators of oral health. A study that administered the Early Childhood Oral Health Impact Scale [14] showed that parent and adolescent perceptions of oral health stemmed from different factors. A study of parent perceptions of their child’s teeth found that fluorosis and demarcated opacities, conditions that affect dental aesthetics, are associated with parental dissatisfaction with appearance, color, and blotchiness [15]*.*

Parent perceptions of their child’s oral health have been shown to be associated with income, education and personal experiences. Mothers with limited schooling tend to have a poorer perception of their child’s oral HRQoL than mothers with higher levels of education. Anxiety and parental distress intersect to influence parental perceptions of their child’s oral health [16]. Lower income parents, who cannot afford health insurance or healthcare, nor gain consistent access to either, tend to view their children’s oral health as worse than higher income parents [17]. In addition, less family income is negatively associated with children and their parents’ oral HRQoL [18]. While previous studies of child and adolescent oral health focused upon parental perceptions of oral health as a concept, few studies have looked at parents’ reports of oral health status specifically focused toward the quantifiable clinical dimension. Moreover, few childhood oral health studies were conducted using qualitative research methods, such as interviews and focus groups [19–21].

A strength of the qualitative approach used in this study, namely focus groups and cognitive interviews, is that it provides a more dynamic picture of the social processes involved than is possible from a static view captured by survey methods. Furthermore, this style of research takes place in natural as opposed to experimental settings, in this study dental practices where children received care, and therefore allows researchers to overcome the discrepancy between what people say and what they do. Qualitative research invariably involves taking the perspective of the participants. A second major feature of qualitative research is that it has a preference for “grounded” concepts and theories. To achieve a grounded approach, however, it is necessary to use, in this case, parents as knowledgeable informants about their child’s physical, social and mental health. The sampling of parents of children with a dental home ensures that they are currently actively engaged in their children’s care. In qualitative research, analysis and collection of data are highly integrated activities. This is necessary to ensure that the research remains grounded. In this study, focus group data suggested dimensions and questions that became important to item development and expert panel review. The subsequent cognitive interviews revealed meanings and surfaced comprehensibility concerns that led to the refinement of items for the field test.

## Methods

This children’s oral health study includes carrying out a systematic review of the literature to identify instruments and survey items associated with oral health status, conducting focus groups to elicit children’s and the parents’ perceptions about the child’s oral health status, drafting items, conducting cognitive interviews to assess children’s and parents’ understanding of the items, revising the items, and then field testing the revised items together with a dental clinical examination. As a PRO measurement study, qualitative research informed a field test survey to assess factors associated with oral health status and the individual’s perceptions and subjective views of these constructs for eventual item development for epidemiological and clinical use. The qualitative methods used in the initial phases of the project are the preliminary steps in the development of oral health item banks and associated short-form surveys for children and adolescents, and their parents. Our oral health model integrates the life-course concept into the dynamics of oral health by including biological, behavioral, and social contexts that change as a person develops through childhood, adolescence, young adulthood, and later adult life. Details of the study, including the model, the development of survey items and the flow of entire study project, are described elsewhere [22]. This paper describes the qualitative work, including focus groups and cognitive interviews, conducted to develop survey items to assess parental perceptions of their children’s oral health status that will be administered in the field test.

### Procedures

We divided children and adolescents into two development age groups: 8–12, and 13–17 years, and conducted four focus groups interviews with them, as described in a previous paper [23], and 29 parents or guardians of children in these age groups from two participating practices. The latter four focus groups probed parent’s attitudes about their children’s oral health and elicited their perceptions using an interview guide displayed in Table 1, and serve, in part, as the basis for this paper. Each focus group was digitally recorded and transcribed verbatim, and entered into NVivo Version 10, a qualitative text analysis database [24]. Word frequencies and content analysis of the focus group transcript, including thematic and narrative analyses, were used to uncover themes. Frequency of mentions pertaining to specific words, for example, provided a sense of what parents viewed as important and what was left out of the group discussion. In our thematic analysis, we identified constructs and dimensions that underlie parental perceptions and found those themes that coalesced into broader categories for use in item development. We reviewed the constructs within each focus group; then, we looked at the total data set, to include all parental responses across the four focus groups and identified six broad domains and, within those domains, numerous dimensions, as shown in Table 2.

**Table 1:** Parent focus group interview guide

Questions	Sub-questions
1. What is the first thing that comes to mind when you hear the phrase “oral health”?	a. ^a^What does “good oral health” mean to you? i. ^b^When you think of your child, would you say that he or she has “good oral health”? ii. ^b^How would you describe a child who has good oral health? iii. ^b^Think about your child’s friends, how would you describe those that have good oral health?
2. What comes to mind when you hear the phrase “poor oral health”?	a. ^a^When you think of your child, what could be thought of as poor oral health? i. ^b^What could be thought as the worst thing for poor oral health? ii. ^b^How would you describe a child with poor oral health? b. ^a^How much of a problem is this among children in your family? i. ^b^How common is it among children in other families who live in this community? Among your friends? ii. ^b^How concerned are you about your son or daughter developing dental problems (dental decay)?
3. As parents, how do you help your child have healthy teeth?	a. ^a^What do you think parents can do (or you as a parent can do) to prevent dental problems in kids (your kids)? b. ^a^As a parent, what do you think are the most important ways to help a child have good oral health? c. ^b^What activities can you do to improve your child’s oral health? d. ^c^What about diet and oral health? e. ^c^What about flouride and oral health? f. ^c^What about the experiences of your other children?
4. Tell me whether you think you can control your child’s oral health?	a. ^b^Are there oral health problems that you feel you can control? i. ^c^What would they be? ii. ^c^What steps can you take to control the problem? iii. ^c^Yourself? iv. ^c^What about other people? Can they do anything to control the problem? b. ^b^When you can control an oral health problem, how does you child respond? c. ^b^When you dealt with an oral health problem, how successful were you? d. ^c^So, you could not control a problem, then what did you do? i. ^b^What was your child’s response? ii. ^b^When you think about it now, does the problem still exist?
5. Other than your family and those who provide care, is there anyone else that you think should or could have an important role in your child’s oral health?	a. ^b^Why? What do you think they can do to help? i. ^c^What about role models in entertainment? ii. ^c^What about advertising and media messages? iii. ^c^What about public policies? iv. ^c^What about insurance?
6. What concerns do you have about your child’s future oral health?	a. ^b^As your child grows older, what oral health problems can affect him/her? i. ^c^How can poor oral health affect a child’s social and psychological well-being? ii. ^c^How can it affect a person’s chances of economic success? iii. ^c^How can it affect one’s general health over a lifetime?
7. Tell me whether you think you can control your child’s oral health?	a. ^b^Are there oral health problems that you feel you can control? i. ^c^What would they be? ii. ^c^What steps can you take to control the problem? iii. ^c^Yourself? iv. ^c^What about other people? Can they do anything to control the problem? b. ^b^When you can control an oral health problem, how does you child respond? c. ^b^When you can dealt an oral health problem, how successful were you? d. ^c^So, you could not control a problem, then what did you do? i. ^b^What was your child’s response? ii. ^b^When you think about it now, does the problem still exist?
8. Other than your family and those who provide care, is there anyone else that you think should or could have an important role in your child’s oral health?	a. ^b^Why? What do you think they can do to help? i. ^c^What about role models in entertainment? ii. ^c^What about advertising and media messages? iii. ^c^What about public policies? iv. ^c^What about insurance?
9. What concerns do you have about your child’s future oral health?	a. ^b^As your child grows older, what oral health problems can affect him/her?

^a^Follow-up

^b^Prompt

^c^Probe

**Table 2:** Focus Group Domains and Dimensions

Domains	Dimensions
Dental Appearance & Attractiveness	Color
	Oral Hygiene
	Diet and Nutrition
	Cigarette Smoking
	How Teeth Look
	Attributions (Why Teeth Look the Way They Do)
	Imputation (Blame/Stigma)
Oral Health Beliefs	Importance of Oral Hygiene
	Importance of Permanent Teeth
	Genetics and Oral Health
	Longevity of Teeth
Good Oral Health	How Teeth Work
	What Healthy Teeth Do
	Association with Child’s Self-Image
	Association with Child’s Self-Presentation
Influences on Good Oral Hygiene	What Parents Tell Their Children About Oral Health
	Influence of Older Siblings
	Influence of Extended Family Members
	Influence of Peers
	Influence of Media Imagery
	Influence of Teachers, Health Professionals
	Influence of Dentists and Dental Treatment
Self-Care and the Life Course	Relation to Good Overall Health
	Relation to Overall Quality of Life
	Relation to Life
	Relation to Higher Education and Career Goals
	Relation to Motivation
	Relation to Happiness
Parental Control	Parental Efficacy
	Sense of Control
	Sense of Helpfulness
	Relation to Child’s Self-Efficacy
	Parental Fear and Anxiety

We identified a preliminary set of items after the focus groups and wrote new items as needed. We administered these items in cognitive interviews with 32 parents or guardians to evaluate their understanding of the meaning of the items, using an interview guide displayed in Table 3. For each item, we probed regarding item content and response options*.* We followed the approach used by DeWalt and colleagues for carrying out PROMIS® cognitive interviews [25]. Specifically, we asked each participant to complete a pencil and paper version of the questionnaire and then a trained interviewer asked questions, or probes, to elicit specific information about each question, including context, time frame and response options. Through “retrospective probing” techniques, we explored how respondents recall information, what time frame they use, and what time frame is beyond their recall. We used intermittent and retrospective probes as part of a “hybrid model” of cognitive interviewing that consisted of a mix of the “think aloud” and “verbal probing” approaches [26]. We elicited parental perceptions of their child’s oral health; recorded their responses; and checked throughout the interview for clarity of the respective items and the parent’s understanding of each item. The draft items were revised based on the cognitive interviews. In sum, through the qualitative analyses of both focus group and cognitive interview data, the research team identified parents’ perceptions about their children’s oral health status.

**Table 3:** Cognitive Interview Guide

Parent-Reported Oral Physical Health		
Oral-Facial Appearance	My child’s teeth are: *Please check all that apply*	Bright white
		Some yellow
		Some green
		Some brown
		Some black
		*How did you decide upon the color of your child’s teeth?*
	My child’s teeth are:	Straight with no spaces
		Straight but with spaces
		Crooked or Crowded
		*What were you thinking about when you selected your answer?*
	When I look at my child’s teeth:	I am happy about how they look
		I feel they could look better
		I am unhappy with the way they look
		*What were you thinking about when you selected your answer?*
	Bad breath for my child is:	Always a problem
		Sometimes a problem
		Never a problem
		*What were you thinking about when you selected your answer?*
Oral Pain	Over the past year, did your child have pain in the teeth or mouth?	YES/NO.
	*If YES, how often?*	S/he had it once or twice over the school year
		S/he has it often during the year
		Sh/e has it every day or almost every day over the school year
		*What were you thinking about when you selected your answer?*
	Where was the pain? *Please check all that apply*	Mouth
		Teeth
		Jaw
		Face
		*How did you decide upon your answer?*
Parent-Reported Oral Mental Health		
Dental Phobia	Did your child ever refuse to go to a dentist because s/he was afraid of: *Please check all that apply*	Getting needles
		Drilling
		Having your teeth pulled
		Having to keep your mouth opened for a long time
		Gagging or choking
		Feeling numb
		Feeling sick
		My dentist
		*What were you thinking about when you selected your answer?*
Dental Anxiety	Did your child ever worry about problems with his/her teeth?	YES/NO
	*If YES, What did s/he worry about?* *Please check all that apply.*	Teeth are going to give him or her pain
		Decayed or rotten teeth
		Teeth that don’t look good
		Teeth that are going to fall out
		Other
		*What were you thinking about when you selected your answer?*
	Have you avoided taking your child to the dentist because of your own concerns?	YES/NO
	*If YES, What were they?*	Use of needles by the dentist
		Drilling by the dentist
		Having the child’s teeth pulled
		Having the child keep his or her mouth opened for a long time
		Gagging or choking by the child
		The child feeling numb
		The child getting sick from the treatment
		I didn’t like the dentist
		*What were you thinking about when you selected your answer?*
Oral Health Beliefs and Behaviors		
Developmental Outcomes	Good oral health is important to my child’s overall health?	Disagree
		Agree somewhat
		Strongly Agree
		*Why?*
	Good oral health is important for my child’s overall happiness?	Disagree
		Agree somewhat
		Strongly Agree
		*Why?*
Longevity	If I don’t help my child care for his/her teeth, his/her life will be:	Shorter by many years
		Shorter by a few years
		About the same
		*Why?*
Life Chances	If my child maintains good oral health, s/he will have a better chance of getting into college and having a successful career.	Disagree
		Agree Somewhat
		Agree Strongly
		*What were you thinking about when you selected your answer?*
Efficacy	By reminding my child to brush his/her teeth, it will help:	Improve his/her oral health
		Maintain his/her oral health
		Won’t make any difference to his/her oral health
		*Why?*
	When my child had a recent oral health problem:	I was able to take care of it myself
		I could not take care of it, and let it go at that
		I could not take care of it, and sought dental care
		My child did not have a recent oral health problem
		*What were you thinking about when you selected your answer?*
	I am able to make a difference to my child’s oral health	Disagree
		Agree Somewhat
		Agree Strongly
		*How?*
	I can do many things to prevent oral health problems in my child.	Disagree
		Agree Somewhat
		Agree Strongly
		*What can you do?*
	Before my child goes to sleep, I remind him/her to brush:	Always
		Sometimes
		Never
		*What were you thinking about when you selected your answer?*
General Well-Being	Thinking about the last week…	
	Has your child been in a good mood?	Never
		Seldom
		Quite Often
		Very Often
		Always
		*Why?*
	Has life been enjoyable?	Not at all
		Slightly
		Moderately
		Very
		Extremely
		*Why?*
	Has your child had enough time for him/herself?	Never
		Seldom
		Quite Often
		Very Often
		Always
		*Why?*
	Has your child had fun with your friends?	Never
		Seldom
		Quite Often
		Very Often
		Always
		*Why?*
Parent-Reported Oral Social Health		
Social Relationships	If my child’s teeth and mouth are unhealthy, his/her friends:	Will still hang out with him/her
		Will probably avoid him/her
		Will definitely avoid him/her
		*What were you thinking about when you selected your answer?*
Social Influences		
Social Comparison - Peers	Compared to other kids my child’s age:	S/he has better oral health
		S/he has the same oral health
		S/he has worse oral health
		*What were you thinking about when you selected your answer?*
Social Network Influences	*In addition to me,* the *most important* influences on my child’s oral health are:	Friends
		Brothers and sisters
		Other family members
		Dentists
		Teachers
		Medical Providers
		*What were you thinking about when you selected your answer?*
	The most important *social* influence my child’s oral health is:	Role models on TV and movies
		Advertising and media messages
		Health education in school
		*Why?*

### Participants

We used patient lists from seven dental practices to recruit parents of children and adolescents (ages 8–17) from dental clinics in Los Angeles County for the focus groups and cognitive interviews. The recruitment sites cover different geographic areas and communities, with diverse ethnic compositions, ranging from low-income underserved, immigrant neighborhoods to high-income professional communities. Institutional review board approval was obtained from the UCLA Office of the Human Research Protection Program (IRB Approval # 13–001330).

### Expert panel review and item revision

The focus group responses permitted us to draw out themes and keywords to understand parents’ perceptions of, experiences with, and beliefs about their children’s oral health. Once themes and narratives from the focus groups were analyzed, together with word frequencies, we conducted thematic and narrative analysis to identify the terms used by the participants*.* Our draft items were: 1) 29 legacy items from original or commonly used measures and instruments: items could be used with both parents and children; 2) 28 legacy items for adults that need revised wording for children, *or* vice versa; and 3) 26 newly written items created as a result of focus group discussions. The initially drafted items were reviewed, revised and approved through an expert panel review; the nine panel members came from backgrounds in public health, pediatric and general dentistry, health services research and oral health status and oral HRQoL measurement.

The newly written draft items were administered in a series of face-to-face cognitive interviews with parents or guardians. We analyzed how parents responded to the interview items with respect to their comprehension, judgment and estimation, and ability to document a response. We also analyzed how parents responded when asked to think about their comprehension to see if their judgments would have changed if the questions were rephrased.

We reviewed all parental comments from the cognitive interviews, identifying item comprehension, word meaning and tense issues, and the degree to which sequential ordering of questions can affect the respondent’s response. In analyzing focus group and cognitive interview responses, we compiled anecdotal evidence from parents to illustrate arguments used by members of the expert panel in their discussion of parental attitudes toward and perception of a child’s oral health.

Upon analysis of the cognitive interviews, items were refined and presented to the expert panel. Based on expert panel review and recommendation, these revised items were combined with existing (“legacy”) items into a field test survey interview.

## Results

### Participants

Table 4 presents the demographics of parents participating in the focus groups (*n* = 29) and the cognitive interviews (*n* = 32). Age, gender, parent marital status, and number of children were not substantially different across the two groups. There were differences in parents’ ethnicity, education, primary language, and family income. Primary language spoken in the home also showed differences with lower numbers of primarily Spanish language speakers in the cognitive interviews and higher numbers of primarily Chinese speakers in the focus group and cognitive interviews. The focus groups had high- and low-income participants with none in the middle-income range ($40,000 to $60,000); while 29% of the cognitive interview participants fell into this middle-income category. These differences are to be expected because the focus groups were held in sites that represented a high-income pediatric dental practice and a low-income community-based dental clinic. The cognitive interview, by contrast, took place in a variety of practice sites.

**Table 4:** Participant Demographics

Variable	Focus Group (*N* = 29)		Cognitive Interview (*N* = 32)	
	Mean	Std Dev	Mean	Std Dev
Age	43.83	6.75	39.59	9.26
	Freq.	Percent	Freq.	Percent
Gender				
Female	22	76%	26	81%
Male	7	24%	6	19%
Total	29	100%	32	100%
Ethnicity				
White	7	24%	10	31%
African American	1	4%	0	0%
Latino	10	34%	17	53%
Asian/Pacific Islander	6	21%	5	16%
Multiracial	0	0%	0	0%
Other	5	17%	0	0%
Total	29	100%	32	100%
Parent’s Education				
Elementary School	2	7%	0	0%
High school or equivalent	2	7%	7	22%
Some College	4	14%	12	38%
College Graduate or higher	16	55%	13	41%
NA	5	17%	0	0%
Total	29	100%	32	100%
Primary Language				
English and other	0	0%	2	6%
English	13	45%	22	69%
Spanish	8	28%	5	16%
Chinese	3	10%	3	9%
NA	5	17%	0	0%
Total	29	100%	32	100%
Parent’s Employment				
Full-time	14	48%	21	66%
Part-time	2	7%	6	19%
Not employed	13	45%	4	12%
Retired	0	0%	1	3%
NA	0	0%	0	0%
Total	29	100%	32	100%
Annual Family Income				
Less than $20, 000	3	10%	1	3%
$20,000 - $29,999	3	10%	6	19%
$30,000 - $39,999	3	10%	2	6%
$40,000 - $49,999	0	0%	6	19%
$50,000 - $59,999	0	0%	3	10%
$60,000 - $69,999	2	7%	1	3%
$70,000 - $79,999	2	7%	0	0%
Over $80,000	10	34%	12	39%
NA	6	21%	0	0%
Total	29	100%	31	100%
Parent’s Marital Status				
Single	2	7%	3	9%
Married	24	83%	25	78%
Not married, living w/ partner	3	10%	1	3%
Divorced	0	0%	3	9%
Total	29	100%	32	100%
	Mean	Std Dev	Mean	Std Dev
No. of children in the home	2.59	0.95	2.63	1.24

### Focus groups

Below, we summarize specific themes emerging from the transcript analysis of the focus group discussions, and corresponding comments that are relevant to children’s oral health item development. We provide quotes to illustrate how concerns are expressed in the parents’ own words. All quotations cited in the text are those of focus group participants (Table 5)*.*

**Table 5:** Key Focus Group Themes with Parents’ Responses

Perception of incidence of tooth color of their child
• “…when they have … discolored teeth more than typically, more maybe like black, dark gray, black and the tooth’s death or missing teeth”
• “I think of white teeth…No nasty yucky stuff that you sometimes see…For my kids, to make sure they do not have yellow”
• “The teeth are not necessarily white in order to have good oral health. The white idea is a new definition of attractive”
• “You have to teach them... I put eggs in coffee and soda. So the egg turned dark. So I told him: ‘Do you want this kind of teeth or do you want this kind of teeth?’ I showed him the two eggs. One was brown and the other one was white. ‘If you don’t brush your teeth, this is the way they will look. You are going to smile and they are going to be brown. If you smiled this way, look, they are going to be white’.”
• “I also see children with black teeth”
Perception of their child’s dental appearance
• “You look at someone with really crooked teeth; I always think, why didn’t your parent do something to help you?”
• “I think even if your teeth are crooked, it is good oral health. It is still healthy. You know. Your teeth are good. They are going to last you for the rest of your life. I think that knowledge is the key; to have people to be informed and with techniques so that they can have good oral health instead of bad oral health”
• “What comes to my mind when I hear the term ‘poor oral health’? I think of crooked teeth.”
Perception of the overall dental appearance
• “I think it is about keeping them clean. And, yes, straighten them and whatever if it is affecting other teeth but I think our aesthetics has gotten in the way of actual health. Do you know what I mean?”
• “I think…big smiling; white”
• “Just for the development, so that they would have a nice smile, good teeth.”
• “For good oral care, maybe that was part of it, but the main thing was so that they had nice teeth: you know. That is all.”
• “Beautiful teeth; beautiful smile”
Perception of frequency of child’s bad breath
• “I think of bad breath…in the back of your throat.”
• “When I think of poor oral health, I say when they have bad breath.”
• “Something we always tell our kids: ‘That is why you have to brush your teeth and your tongue if you do not want to have bad breath’; because when you talk to people, you know, socially… being older, again, you kind of step away and move away from people who have bad breath or do not have good oral health.”
Perception of child’s fear of dental treatment
• “I had three daughters. They were always afraid because my parents always kept saying if you don’t brush your teeth, (you are) going to go to the doctor and (it) is going to hurt you. So they didn’t want to go to the dentist because they were afraid of the treatment that could be painful. And in reality, it is painful.”
• “I am honest with my kids. I told them, it is going to hurt but is not going to kill you. That is the experience with the kids. I made a rule; I am not going to lie to you. It is going to hurt but it is not going to kill you. Each child is different based on the level of fear.”
• “My son does not have a fear of the dentist. He has a fear of needles…I think he is motivated from not wanting to go and get fillings or having the shots.”
Perception of child’s worry over dental problem
• “If you do not have a good oral health as an adult, you will get cavities, your teeth will fall out. You would have periodontal disease. I mean, those are the things that I worry about if they do not access. I do believe that if I take good care of her teeth, up ‘til 18, which is when most of her teeth were growing and forming, and we are good on their teeth, I am hoping that would give them consideration that will last for a lifetime because they will have good set of starting points. Growing adult teeth are going to be (a) higher quality of enamel and not have cavities because again from my experience that was true for me.”
• “You have to spend time as an adult to take care it all, and if I can give them the luxury of not having to worry about it and not having to take care of it, why wouldn’t I?”
• “Well, I took mine when he was 2 years; the other one was 3 years. My main purpose was not for their teeth at that time. It was so that they would have no fear. So that they would be happy and perceive it as a positive. That was very important to me; that they not be afraid to go to the dentist or be afraid of the dentist because we still, me and my husband, are still afraid. Me, my husband, my sisters have a big fear.”
Attitude toward the importance of child’s oral health to overall health
• “I have heard, too. Actually, one of my coworkers told me, and it stuck to my head that if you do not clean your teeth, you get sicker faster because more germs build up. I kind of took that seriously. He is kind of right. Now, has this been proven, studied? I do not know, but I truly believe that if you take care of your teeth, you do not get sick as often as most people do.”
• “When I hear oral health what comes to mind for me is the whole body.”
• “At 13–14 years old, I did not have a good oral health and I have cavities in my adult teeth; we all end up dealing with that as an adult. And I am not even talking about cardiovascular disease.”
Attitude toward the impact of their care for child’s oral health on longevity
• “I took a class in college…I remember them saying that if you do not take care of your teeth, it takes off—I do not remember the number— like 5 or 6 years from your life. I remember that affecting my idea of taking care of my teeth.”
Attitude toward oral health maintenance and life chances.
• “I look at it as an investment. Until they are 18, I am investing in their teeth, because I am hoping that their teeth are much more healthy than mine.”
Attitude toward their capacity for support of their child’s oral health prevention
• “I agree but it seems like, they say that plaque builds up even if you brush every day. It builds up within a period of 6-month. So, if you do not go to the dentist every 6 months, you are not getting that extra build up taken off. So, it does not matter how much you are brushing your teeth. That can add up.”
• “If you are maintaining a structured situation at home with your kids, making sure that they are brushing and flossing on a regular basis, it will not be that costly. There will just be cleanings, and maintenance to make sure they are okay, but if they are not brushing and flossing, that is when you get into caries, cavities, fillings, and other issues. That can be costly as a result of not having home discipline.”
• “My mother had dentures in her 20s because she had a gum disease called pyorrhea. So, for me, I am on it because I do not want it to happen to me or my children because of laziness or for being unaware of the details or of all the things that have to be taken care of as far as we said. Things such as scraping your tongue, flossing, brushing, and making that a routine. This is very important in my house.”
• “Setting a schedule. Being available to make the time to stand there. Say, hey, go brush your teeth and leave it up to the kid. If you know that you have a child that will not do it on their own, you have to make the time to stand there and either do it for them, do it with them or instruct them how to do it.”
• “Also, maybe the kids need to see the parents do it. Sometimes kids don’t do what we tell them. They do what they see. You know what I mean. If they see their parents brushing their teeth; that may mean that you have to do it four times instead of two. Parents need to do it at different time frames so that when the kids see you, they would say, mommy is doing it, maybe I’ll do it”
• “It also has to do with the way you are raising your kids in terms of cleanliness. I only have one child. We are always telling her to brush her teeth in the morning and in the evening. Now, because we pushed her since she was small, I do not have to push her to wash with the mouthwash. It becomes a good habit. In order for this to become a good habit, we have to teach them to do it.”
• “Do they floss as often as they would like? No. Do they brush twice a day? Yes. Do they do a good job? Uh. So, relatively speaking, yes. The reason why I take them to the dentist every 6 months is so that even if they do a good job as far as brushing their teeth twice a day... So, you know. Do they do it on their own if I do not remind them? My 10 year-old, no.; my 12 year-old, yes. He has a better attitude. As she gets older, maybe the fact (that) when the peers smile and she notices.”
Parent’s capacity to influence their child’s oral hygiene behavior
• “Well yeah, over the years, I have done different things with my kids, like my son: I have to put little notes up on the mirror to remind him what to do, they share a bathroom so I would buy both mouthwash so they have their”
• “I make sure he brushes his teeth before he goes to bed, after taking a shower every night.”
• “You just have to remind them. I never brush his teeth. As a toddler, I did. He has always done it, but if I do not remind him, he probably won’t do it. It is about reminding him and making the dental appointments.”

### Oral-facial appearance

Dental appearance was viewed by the parents in terms of color of the teeth and also by the straightness of the teeth as in the following comment: “You look at someone with really crooked teeth; I always think why didn’t your parent do something to help you?…” There were also differences between high- and low-income parents with respect to perception of tooth color. This aspect of class and culture is found in the perception of “bright white” versus “white” teeth. High-income parents equated the value of bright white teeth with their ability to employ professional services to brighten teeth. Some indicated that this was a matter of style and, for this reason, had their children’s teeth brightened. One parent remarked: “The teeth are not necessarily white to have good oral health. The white idea is a new definition of attractive.”

### Dental phobia and anxiety

An area of parental concern is their child’s fear and worry over dental treatment; one parent said: “it was very important to me that they not be afraid to go to the dentist because we, me and my husband, are still afraid,” indicating they do not want to perpetuate dental anxiety in their children.

### Support for oral health prevention

In many instances, the parents also discussed their capacity to support their child’s oral health prevention and its economic implications. One parent remarked: “If you are maintaining a structured situation at home with your kids, making sure that they are brushing and flossing on a regular basis, it will not be that costly.”

### Oral health and systemic health

In their responses, parents viewed oral health through a holistic lens, namely, as a way to stay healthy, overall. One parent said: *“*I have heard, too. Actually, one of my coworkers told me, and it stuck to my head that if you do not clean your teeth, you get sicker faster because more germs build up. I kind of took that seriously. He is kind of right. Now, has this been proven, studied? I do not know, but I truly believe that if you take care of your teeth, you do not get sick as often as most people do.”

### Oral health and the life course

Parents viewed oral self-care in terms of both maintaining general overall health, and living a longer life as a result. One parent said. “I took a class in college…I remember them saying that if you do not take care of your teeth, it takes off—I do not remember the number— like 5 or 6 years from your life. I remember that affecting my idea of taking care of my teeth.”

### Cognitive interviews

We identified a number of issues from the cognitive interviews that informed subsequent item development*.*

### Wording

A concern was the confusing wording of some of the draft items. In assessing their child’s teeth, some respondents had a difficult time answering the question about tooth color, because they may perceive teeth as neither “bright white” nor “yellow,” but as other shades, as represented on standard shade guides. The term, “white,” indicates pale or free of color, and opaque; while “bright” denotes a radiant, shining and sparkling appearance; “yellow,” by contrast, indicates discoloration. We tried to determine how respondents communicated these shades and to then address the ways the revised survey questions could address this confusion. The focus group analysis identified that color was important; however, when this was explored with parents in the cognitive interviews, the issue turned out to be quite complex and not a mono-dimensional construct.

Some parents were confused when asked to choose between the terms, “maintain” through proper oral care and diet to prevent oral disease, and “improve” to deter progression of oral conditions, such as dental caries, through treatment. Parent respondents did not understand that if the child already has active oral disease, then it is a question of improvement. However, if the child does not as yet have active disease, then the parent is helping the child maintain good oral health and thus prevent the onset of new disease.

### Temporal context

The temporal dimension was another concern. Certain items required parents to recall a time when they were concerned about a recent oral health problem or indicate any instances of dental anxiety or phobic reactions. Parental responses varied, with some respondents solely commenting on their child’s current status, while others talked about a previous situation, or remarked on gradual improvements or worsening of specific conditions. We also found that seasonality (i.e., time of the year when an interview took place) affected parental attitudes. Interviews conducted during the school year may have affected parents’ perception of their children’s overall well-being, with many parents expressing a harried pace of being engaged in the urgency of their school responsibilities; interviews conducted during school breaks and summer vacations found parents commenting that their children are happier, as these times provide a more relaxing schedule.

### Item revision

Based on the themes and dimensions that surfaced throughout qualitative work, we undertook item revision. In reviewing parent responses to cognitive interview questions, the expert panel suggested revisions to certain items in advance of survey administration (Table 6)*.*

**Table 6:** PROMIS Parent Survey of Child Oral Health Revised Items and Reasons for Revision

Cognitive Interview Items	Cognitive Interview Revisions	Field Survey Items
6a) My Child’s Teeth Are: Bright white Some Yellow Some Green Some Brown Some Black	Some parents did not know how to report the color of their children’s teeth and were confused by “bright white”; a couple of parents had their children’s teeth brightened by dentists, but most of the others felt either “white” or “yellow” was more like the color; however, some had a brown tooth among the other colors. Given the option to include different colors seemed to solve the problem.	The color(s) of my child’s teeth are (check all that apply): Some bright white Some white Some yellow Some green Some brown Some black Total
6b) My child’s teeth are: Straight no spaces Straight with spaces Crooked or Crowded	The following comments raised the issue of upper arch vs. lower arch; this could add information, but complicates the response. However, allowing parents to respond to multiple individual items such as: straight, spaces, crowding and crooked, would increase the respondents’ ability to characterize aesthetic and occlusal aspects. The “straight with spaces” response is less important in the younger ages than in the adolescent. Responses included: “many are straight but there are some crooked, but none are crowded”; “straight but with spaces for the upper teeth and crooked and crowded for the lower teeth”; “child’s teeth were crooked or crowded and at the same time there were spaces.”	My child’s teeth (check all that apply): Are straight Have spaces Are crooked Are crowded Total
6c) When I look at my child’s teeth: I am happy with how they look I feel that they could look better I am unhappy with the way they look	Parents were either happy or felt they could look better, mainly because of teeth that are crooked or crowded, to a lesser degree because of color or cavities. We changed the response from “happy/unhappy” to “fine”/“a little better”/“a lot better.” Responses included: “They could look better because of crooked teeth”; “they could look better because they were crooked and had spaces”; “they could look better because of cavities in primary teeth”; “they could look better because of crowding and color”; “they could look better after the braces close the spaces.”	When I look at my child’s teeth: They look fine They could look a little better They could look a lot better Total
6d) Bad breath for my child is: Always a problem Sometimes a problem Never a problem	Most parents did not think this was a problem. Some made reference to “morning breath,” i.e., “in the mornings when she wakes up”; or “when he gets home from school.” One parent said: “Always has bad breath” We added a question to address parents’ concern about their child’s bad breath: ‘How often does your child have bad breath? Another item was added that focuses on parental concern’.	How often does your child have bad breath? Always Almost always Often Sometimes Almost never Never Total
	Originally, this was one item in the cognitive interview; however, we chose to create two separate survey items to better explain which parents noticed the problem and their level of concern.	How often are you concerned about your child’s bad breath? Always Almost always Often Sometimes Almost never Never Total
6e) Did your child ever refuse to go to a dentist because s/he was afraid of: Getting needles Having your teeth pulled Drillings Having to keep your mouth opened for a long time Gagging or choking The child feeling numb I didn’t like the dentist The child getting sick from the treatment	The temporal issue was resolved by asking parents to respond in terms of the present rather than “ever.” The wording “refused” confused the issue, because it is the parent’s responsibility for accessing care; the question was changed from “refused” to “being afraid of.” The term, “refuse,” gives the child agency, whereas “afraid” is amenable to parents talking about fear. We do not wish to imply that the child has autonomy or the freedom to dictate dental treatment or refuse care. Responses included: ‘Getting needles, drilling, gaging, my dentist’ “when 4 years old went to a dentist who wasn’t good with children, now fine”; “the dentist wasn’t good, the present one is”; and “not a problem now” ‘checked getting needle and drilling’	What is your child afraid of? (Check all that apply) None selected He/she dislikes needles He/she is afraid of having teeth pulled He/she dislikes drilling He/she dislikes keeping my mouth opened for so long He/she is afraid of gagging or choking It was a new experience He/she dislikes feeling numbness He/she is afraid of the dentist He/she is afraid of feeling sick He/she is afraid of the mask and glove Total
6f) Did your child ever worry about problems with his/her teeth? Yes: What did s/he worry about? No Teeth are going to give him or her pain Decayed or rotten teeth Teeth that don’t look good Teeth that are going to fall out Other	The new version allowed for parents to check all that applied and added “pain with teeth,” rather than only “teeth” and “bad breath.” Parent responses included: “decayed teeth and decayed that don’t look good due to color”; ‘decayed and rotten teeth and teeth that don’t look good’; “decayed and rotten teeth”; ‘getting needles, drilling, gagging and my dentist’; and “when was four old went to a dentist who was good with children, now fine.”	Does your child worry about problems with his or her teeth? Yes/No What does he or she worry about? Check all that apply. (Check all that apply) Yes (see responses below) No Pain with his/her teeth Decayed or rotten teeth His/her teeth don’t look good His/her teeth are falling out Bad breath Other, please explain Total
6 g) Good oral health is important to my child’s overall health. Disagree Agree somewhat Strongly agree	Many parents believed that there is a link between poor oral health and systemic disease. The question was not changed in this case. The response categories were increased by adding "disagree somewhat," which provides more balance.	Good oral health is important to my child’s overall health. Disagree Strongly Disagree Somewhat Agree somewhat Agree Strongly Total
6 h) If I don’t help my child care for his/her teeth, his/her life will be: Shorter by many years Shorter by a few years About the same	The comments regarding teeth and longevity came from several Latino parents who saw a direct link between the number of teeth and the years of life. This link opened our eyes to the issue of health over the life course. The question was put in the positive and the response was made comparative; rather than a simple “shorter by many years,” we changed it to “many more years than if I didn’t take care of them”	If I help my child care for his or her oral health, he or she will live: Many more years than if I didn’t take care of them Somewhat more years than if I didn’t take care of them The same number of many of years than if I didn’t take care of them Some fewer years than if I didn’t take care of them Many fewer years than if I didn’t take care of them Total
6i) If my child maintains good oral health, s/he will have a better chance of getting into college and having a successful career. Disagree Agree somewhat Agree strongly	Parents found this question confusing because they did not view oral health as important to getting into college. They saw that process as impersonal, namely relying on data, such as the GPA, SAT/ACT scores and recommendation letters, and not the direct contact with the admissions decision-makers. By contrast, parents regarded a career as a personal process involving direct interaction where oral health plays a more important role. As a result, we decided to relate oral health to chance of success in life.	If my child maintains good oral health, he or she will have a better chance of success in life. Disagree strongly Disagree somewhat Agree somewhat Agree strongly Total
6j) When my child had a recent oral health problem: I was able to take care of it myself I could not take care of it myself and let it go at that I could not take care of it, and sought dental care My child did not have a recent oral health problem	This was not an open-ended question, but one that is factual.	During the last 12 months, did your child have an oral health problem Yes (check all that apply) No I was able to take care of it myself without going to the dentist I did not do anything about it I took my child to the dentist Total
6 k) I am able to make a difference to my child’s oral health Disagree Agree Somewhat Agree Strongly	Rather than using the term, “make a difference,” efficacy was better reflected by the action term “prevent oral health problems”; two responses, “Disagree strongly” and “Disagree somewhat” replaced “Disagree,” to provide more balance in the response categories.	I can do many things to prevent oral health problems in my child. Disagree Strongly Disagree somewhat Agree somewhat Agree strongly Total
6 l) Before my child goes to sleep, I remind him/her to brush: Always Sometimes Never	We changed the question to address our expert panel, whose members preferred a conceptual approach, rather than a strictly behavioral one. The previous version asked the extent to which the parent had to remind the child (behavior), while the revised version asked about the parent’s belief in the effectiveness of reminding the child to brush. This necessitated a change in responses to: “…make his/her oral health better”; “…keep his/her oral health the same”; ‘…make no difference in his/her oral health’.	By reminding my child to brush his or her teeth, I believe: It will make his/her oral health better It will keep his/her oral health the same It will make no difference to his/her oral health Total

### Oral-facial appearance

The first set of revised items (6a-6d) deals with the parent’s perception of physical state of their child’s oral health. Some parents did not know how to report the color of their children’s teeth and were confused by “bright white”; a couple of parents had their children’s teeth brightened by dentists, but most of the others felt either “white” or “yellow” was more like the color; however, some had a brown tooth among the other colors. Given the option to include different colors seemed to solve the problem.

### Dental phobia and anxiety

The second set of revised items (6e-6f) refers to parental perception of their child’s feelings about the dental experience, When parents were originally asked, “Did your child ever refuse to go to the dentist because s/he was afraid of…” various routine clinical procedures, the wording “refused” confused the issue, because it is the parent’s responsibility for accessing care; the question was changed from “refused” to “being afraid of.” The term, “refuse,” gives the child agency, whereas “afraid” is amenable to parents talking about fear. We do not wish to imply that the child has autonomy or the freedom to dictate dental treatment or refuse care.

### Oral health and the life course

The third set (6 g-6i) of revised items concerns parental attitudes about the value they place on oral health as it affects the child’s life course. Parents were originally asked, “If I don’t help my child care for his/her teeth, his/her life will be…” and select from the following responses: “Shorter by many years; Shorter by a few years; About the same.” Comments regarding teeth and longevity came from several Latino parents who saw a direct link between the number of teeth and the years of life. This link opened our eyes to the issue of health over the life course. The question was put in the positive and the response was made comparative; rather than a simple “shorter by many years,” we changed it to “many more years than if I didn’t take care of them.”

Parents were originally asked to agree or disagree with the following: “If my child maintains good oral health, s/he will have a better chance of getting into college and having a successful career.” Parents found this question confusing because they did not view oral health as important to getting into college. They saw that process as impersonal, namely relying on data, such as the grade point average, SAT/ACT scores and recommendation letters, and not the direct contact with the admissions decision-makers. By contrast, parents regarded a career as an interpersonal process involving direct interaction where oral health plays a more important role. As a result, we decided to relate oral health to chance of success in life.

### Parental efficacy

The final set (6j-6 l) of revised items addresses the parent’s sense of efficacy, specifically a sense of their own control over their children’s oral health behaviors. We asked parents to think about the following question, “By reminding my child to brush his/her teeth, it will help…” and select from the following responses: "1) Improve his/her oral health; 2) Maintain his/her oral health; and 3) Won’t make any difference to his/her oral health." We changed the question to address concerns of our expert panel, whose members preferred a conceptual approach, rather than a strictly behavioral one. The previous version asked the extent to which the parent had to remind the child (behavior), while the revised version asked about the parent’s belief in the effectiveness of reminding the child to brush. This necessitated a change in responses to: "1) make his/her oral health better; 2) keep his/her oral health the same; and 3) make no difference in his/her oral health."

## Discussion

The National Institutes of Health promote the use of PROMIS® and other measures for research and in clinical practice through the “Person-Centered Assessment Resource” [27]. There is an extensive library of item banks, but an oral health item bank is not yet available. This paper described the type of qualitative work needed towards filling this gap.

We reviewed the literature, conducted focus groups, drafted items, and evaluated them in cognitive interviews in order to revise them for a forthcoming field test. Using the focus groups and cognitive interviews as formative research methods we set out to: 1) document the experience of oral health as seen by children and adolescents across physical, mental and social health domains; and 2) gain understanding of the effects of oral health attitudes, values, beliefs, and practices of the parents or guardians across these domains, which is the focus of this paper.

### Implications for PRO measurement

Our approach combines formative research methods with survey research to provide a more dynamic picture of child and adolescent oral health status than is possible from a static view captured by survey methods, alone. Our study is strengthened by including children with ethnic and geographic diversity and parents/guardians from a variety oral health professional settings. Mixed methods research holds the promise of expanding the availability of appropriate oral health status PRO measurement tools, especially those linked directly to comprehensive clinical measures of oral health status.

To date, most child oral health studies using qualitative methods focused on how parent reports can inform the planning and the design of preventive interventions. Several studies describe the use of qualitative methods with parents relative to predisposing factors leading to childhood caries. Three studies utilize the Fisher-Owens conceptual model of child, family and community influences on oral health outcomes of children in terms of caries [28]. Duijster et al. [29] collected data from focus group interviews with predominately immigrant families in the Netherlands to understand parental views on the influences on children’s oral health behavior and to then develop caries prevention programs for this population. Riggs et al. [30] used focus groups and semi-structured interviews with immigrant and refugee families in Australia to guide the design of culturally competent caries preventive interventions for migrant-serving agencies. Cortes et al. [31] used focus groups and semi-structured interviews with recent Latino immigrant families to understand their perceptions and experience with dental care on behalf reducing oral health disparities. Two studies address communication about early childhood caries prevention and intervention between program staff and family enrollees in publicly funded programs. Mofidi et al. [32] conducted focus groups with Early Head Start staff, parents and pregnant women in North Carolina to inform the design of theory-based educational interventions. Kelly et al. [33] conducted focus groups with low-income Medicaid recipients in Kentucky to surface barriers to early children caries prevention and care. These studies have a limited view of oral health status as their objective since their focus is on the prevention and treatment of early childhood caries. By comparison, the COHSI is a more extensive view of children’s oral health, including occlusal and facial characteristics, and therefore embodies a broader view of child and adolescent oral health that takes into account aesthetic considerations. What PRO measurement lends to this outcome, linked directly to a clinical index of oral health status, is a formalization of the inputs across the three domains, namely physical, mental and social health.

Together, focus groups and cognitive interviews deepened our understanding of lay insights about oral health, from the parent’s perspective. These qualitative methods in our formative research activities also provided a look into parents’ perceptions along cultural and class lines. For some low-income Latino parents, each tooth lost represented a loss of a year of life. By contrast, upper income parents were concerned that disease in the mouth can cause systemic disease; therefore having poor oral health represented suboptimal overall health and wellbeing. Both groups believed that poor oral health has implications for the life course, but better off parents assumed that preventive oral health behavior will directly result in healthier children now and in the future. Parents admitted that their own fears and anxieties about dental treatment were a stimulus for assuring that their child receives dental care; although they may not have been aware that these emotions can be subliminally projected onto their children. It would be interesting to see whether children whose parents experience fear or anxiety about the dental visit carry similar emotional burdens when they present for dental care.

In sum, our research has the potential of broadening the concept of oral health, thereby informing PRO measurement, by offering sets of parent items that encompass the domains used in the PROMIS® methodology. At the same time, this research moves toward enhancing an understanding of the link between perceived and clinically determined oral health.

## Conclusions

The paper reports on discussions and interviews with the parents or guardians of children and adolescents, ages 8–17, a subgroup that, in addition to changes associated with emerging-developmental skills and functions, experiences transitional and permanent dentition, and relatively high rates of dental and occlusal problems. This study has several limitations. First, our sample is comprised only of children who are already in dental care and their parents or guardians who agreed to participate in a study to understand the perceptions of parents whose children have a dental home. In future studies, we will interview parents of users and non-users of dental services to see whether there are any differences in their perceptions of oral health status. Second, we did not measure health literacy in this phase because we drew upon the responses of a wide range of parents in developing items for the cognitive interview and the subsequent field test. Through the cognitive interviews with parents, in an informal way, we were able to get a sense of the comprehensibility and meaning of the various items developed from focus groups. Third, parents’ perceptions involved in the evaluation and refinement of items were inherently subjective, reflecting observations of their children’s oral health, and this was the primary interest of our study. Our intent was specifically to gain a sense of the parent’s understanding of their child’s oral health, as a concrete example of oral health status, and not as a general concept, because, eventually, we wished to relate these responses to the clinical oral health of their child in the field test.

Qualitative methods and subsequent thematic and narrative analyses uncovered key lay-oriented dimensions, notably the relationship between oral health, systemic health and the life course. In the cognitive interviews, parents entered multiple responses to questions related to the look of their child’s teeth, and their overall perception of tooth color. Parents also assessed their child’s fear or discomfort with the dental experience, and other social and psychological concerns related to oral health status. Additionally, the temporal dimensions of certain items were specified; for example, oral pain and mood items were revised to include duration of the symptom or mood state. The qualitative methods used in the initial phases of the study will lead to the development of oral health item banks for children and adolescents and collect data not possible through other methods. The oral health items that will be finalized following the field test can be used by dentists, oral health researchers and professionals, and public policy makers for oral health screening, program assessment, oral health evaluation with large populations as well as oral health management and policy planning.
